# Metabolomic Profiles on Antiblastic Cardiotoxicity: New Perspectives for Early Diagnosis and Cardioprotection

**DOI:** 10.3390/jcm11226745

**Published:** 2022-11-15

**Authors:** Luca Fazzini, Ludovica Caggiari, Martino Deidda, Carlotta Onnis, Luca Saba, Giuseppe Mercuro, Christian Cadeddu Dessalvi

**Affiliations:** 1 Department of Medical Sciences and Public Health, University Hospital of Cagliari, Strada Statale 554, Km 4.500, 09042 Monserrato, Italy; 2 Department of Radiology, Azienda Ospedaliero Universitaria (A.O.U.), di Cagliari—Polo di Monserrato, Strada Statale 554, 09042 Monserrato, Italy

**Keywords:** cadiotoxicity, cardioprotection, metabolomics, heart failure

## Abstract

Antiblastic drugs-induced cardiomyopathy remains a relevant cause of morbidity and mortality, during and after chemotherapy, despite the progression in protective therapy against cardiovascular diseases and myocardial function. In the last few decades, many groups of researchers have focused their attention on studying the metabolic profile, first in animals, and, subsequently, in humans, looking for profiles which could be able to predict drug-induced cardiotoxicity and cardiovascular damage. In clinical practice, patients identified as being at risk of developing cardiotoxicity undergo a close follow-up and more tailored therapies. Injury to the heart can be a consequence of both new targeted therapies, such as tyrosine kinase inhibitors, and conventional chemotherapeutic agents, such as anthracyclines. This review aims to describe all of the studies carried on this topic of growing interest.

## 1. Introduction

The life of cancer patients can be threatened by both malignancies and cardiotoxicity (CTX) derived from anticancer therapies. Therefore, in recent years, CTX has become one of the main challenges for cardiovascular research to reduce the morbidity burden in long-term cancer survivors.

Anthracyclines are, at present, the most studied antiblastic drugs [1], however, many others have shown the ability to determine CTX, caused mainly by their joint action on both the signaling pathways required for tumor growth and those involved in normal cardiovascular function [2].

The manifestation, severity, duration, and type of CTX vary based on malignancy and anticancer therapy. Regarding the range of possible cardiovascular manifestations of CTX, heart failure (HF), arrhythmias, arterial and venous thrombotic events (i.e., acute myocardial infarction, pulmonary embolism), vascular diseases (arterial hypertension, aortic dissection), myocarditis and pericarditis constitute the most common [3]. Among cancer survivors, the risk of developing cardiovascular disease as a result of antiblastic-induced CTX is higher than the risk of a recurrence of the tumor [4]. Moreover, among breast cancer survivors, mortality resulting from heart disease is higher than that of breast cancer itself [5]. In light of this evidence, the early identification of CTX is a central topic in cardiovascular diseases.

Unfortunately, conventional cardiovascular diagnostic tools often show detectable changes only after cardiac damage development [6]. Accordingly, in recent decades, basic and translational research has focused on the mechanisms of CTX, intending to identify the development of CTX at early stages.

## 2. What’s Metabolomics?

In this setting, omic sciences, such as genomics, transcriptomics, proteomics, and metabolomics, aim to provide an integrated explanation of different biological systems. Metabolomics is the science that analyzes all the metabolites produced by enzyme reactions that interact with each other, constituting human metabolism. Studying metabolites allows us to describe a detailed metabolism profile (the metabolome) of a cell, a tissue, or an organism. Some metabolites need time to register significative variations in human biological fluids, and they could stand for old variations or predict future conditions and diseases.

**Figure 1:**
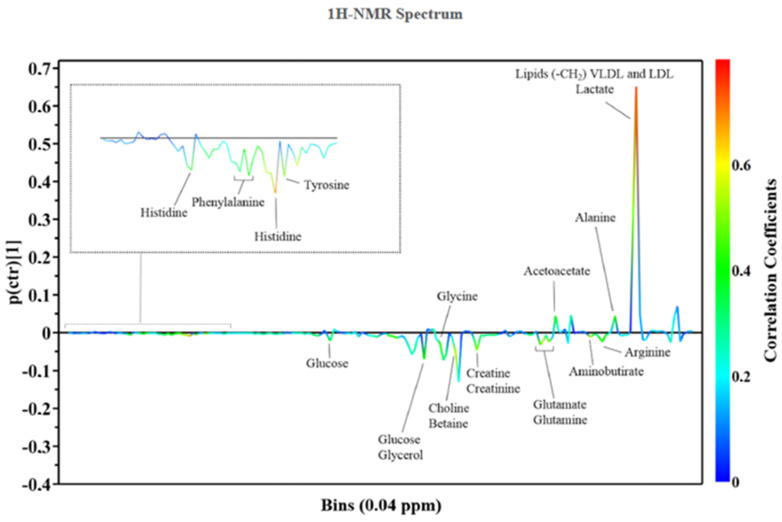
Metabolic pattern from 1H-NMR Spectrum. Central illustration. The continuum of the development of cardiotoxicity due to anticancer therapies and the potential application of metabolomics to its investigation and diagnosis.

The metabolomic analysis uses analytical technologies to identify low-molecular-weight molecules such as amino- and fatty-acids, vitamins, nucleic acids, organic acids, lipids, carbohydrates, polyphenols, and inorganic and elemental species. Biological samples provide a metabolic profile that differs depending on the specimen under investigation and the analytical technique used [7] (Figure 1).

Metabolomics analysis usually follows a protocol composed of three steps:

1. Sample collection and storage;
2. Sample analysis, using nuclear magnetic resonance spectroscopy and mass spectrometry techniques;
3. Data analysis using multivariate statistical tools.

The Human Metabolome Database [8] stores over 40,000 metabolites that are recognized, quantified, and classified by the Human Metabolome Project [9].

Search methods: We searched on Pubmed library till August 2022 using the search terms “cardiotoxicity” AND “metabolomic*” or “metabonomic*”. Two review authors independently performed study selection and data extraction.

## 3. Until 2019: Early Detection of Drug-Induced Cardiotoxicity in Animal Studies

Since the early 2000s, many researchers have conducted metabolomic studies on animal models to describe cardiotoxicity (CTX) and cardioprotection metabolic profiles (Table 1).

Andreadou’s group, analyzing Doxorubicin (DOX)-treated excised rat’s heart by H-Nuclear Magnetic Resonance Spectroscopy (H-NMR), found that succinate and acetate could predict CTX and could represent biomarkers. According to these findings, a phenolic antioxidant, such as oleuropein, with well-known cardioprotective effects, could prevent or reduce CTX [10]. Some years later, they carried out an additional study, which analyzed metabolomic data with echocardiography and histopathology cardiac findings, inflammatory cytokines, nitric oxide homeostasis enzymes (iNOS, eNOS), and apoptosis enzymes (AMPK). They established the putative role of oleuropein in CTX prevention; oleuropein-induced AMPK activation and iNOS suppression can avoid DOX-induced CTX [11].

During the same period, Tan’s group described twenty-four metabolites involved in citrate cycle, glycolysis, amino acids, and lipids metabolism associated with DOX-induced CTX in rats [12].

At that time, pirarubicin (THP), a DOX analog with excellent activities against acute leukemia, lymphomas, and some solid tumors, was also studied; due to its cumulative CTX risk, a novel THP liposome powder formulation (L-THP) was developed. Cong and his group analyzed the urine metabolic trail of Sprague-Dawley rats after three consecutive doses of L-THP or free THP (F-THP). CTX was clinically assessed for a significant bodyweight decrease in treated versus control rats and confirmed their results through histopathology findings. The metabolomic analysis found that L-THP produced minimal metabolomic changes compared to F-THP. However, THP doses led to severe metabolomic alterations in energy production pathways such as citrate, lactate, D-gluconate-1-phosphate, and N-acetyl-DL-tryptophan significant downregulation [13].

At the same time, other groups of researchers focused their attention on the optimization of biomarkers for the early prediction of CTX based on metabolomics, such as Li and Schnackenberg’s groups; in the case of the former, they found ten CTX particular metabolites of whch L-Carnitine, 10-hydroxydioxycortic acid and lysophosphatidylcholine. These metabolites changed through metabolomic analysis with ultra-performance liquid chromatography–quadrupole time-of-flight mass spectrometry (UPLC–Q-TOF-MS), before biochemical and histopathological disorders were detected [14]. Moreover, Schnackenberg’s group designed an NMR spectroscopy and a mass spectrometry-based metabolomic study to recognize early biomarkers of DOX-induced CTX in male mice. A 3 mg/kg DOX dose or saline was dispensed weekly for 2, 3, 4, 6, or 8 weeks. The authors found a rise in 18 amino acids and four biogenic amines after a 6 mg/kg cumulative dose. Myocardial lesions were identified only at a cumulative dose of 18 mg/kg and significant cardiac damage was identified at 24 mg/kg cumulative dose. Plasma concentrations of succinate and lactate were significantly modified after a 6 mg/kg cumulative dose [15].

In 2016, another research group collected and analyzed rat plasma samples using UPLC–Q-TOF-MS after cyclophosphamide (CY) administration on days one, three, and five. The authors found altered concentrations of several metabolites (involved in linoleic acid and glycerol phospholipid metabolism) in the CY-treated group compared to the control group [16].

During the same year, Chaudhari’s group used H-NMR to analyze the culture medium of human-induced pluripotent stem cell-derived cardiomyocytes, exposed to DOX for two days or six days. Repeated exposures led to the reduced consumption of acetate and pyruvate with an increase of formate, while a single exposure to DOX did not cause those adaptations. The authors concluded that acetate, pyruvate and formate could be proposed as biomarkers of DOX-induced CTX [17].

One year later, Quan Jun’s group conducted a metabolomic study to identify the protecting role of dexrazoxane (DZR) against DOX-induced CTX: mice were randomly divided into two groups (tumor and control), each divided into four treatment subgroups (control, DOX, DZR and DOX plus DZR). DOX treatment was characterized by a surge of 2-hydroxybutyrate, 5-hydroxylysine, 3-hydroxybutyrate and 2-oxoglutarate levels and a reduction in glutamate, glucose, acetone, cysteine, methionine, isoleucine, aspartate and glycylproline levels. On the other hand, administration of DZR determined an increase in 3-hydroxybutyrate, lactate, glutamate, and alanine levels and a reduction in trimethylamine N-oxide, glucose and carnosine levels. The authors highlighted that all of these metabolic alterations seem to be related to CTX pathways [18].

The role of periplocymarin on DOX-induced CTX has not been studied yet. Yun’s group randomized C57BL/6 mice into three groups (the control group, DOX-treated, and DOX plus periplocymarin). Liquid chromatography (LC) and mass spectrometry (MS) analysis showed that DOX causes an increase in serum ceramide, and that pre-treatment with periplocymarin could avoid this accumulation. They stated that these results suggested that periplocymarin moderated cardiomyocyte apoptosis, protecting myocytes from DOX-induced CTX; moreover, the “de novo” synthesis of ceramides appeared to be involved in this process [19].

In 2020, another research group directed their attention to assessing the early detection of DOX-induced CTX by evaluating cardiac metabolic signature with hyperpolarized NMR. Timm’s group used hyperpolarized NMR to assess metabolic fluxes in vivo, showing that alterations in oxidative mitochondrial carbohydrate metabolism, measured by hyperpolarized NMR, clinically preceded detectable DOX-induced heart failure in a rat model. Male rats, divided into three groups, were treated with either a sterile saline (control group), a low dose of DOX, or a high DOX dose for five successive weeks. The high DOX-dose group significantly improved cardiac troponin I and lactate dehydrogenase (LDH) levels. Assessing cardiac function by CINE-Magnetic Resonance Imaging (MRI) at weeks one, three, and six, they found that the left ventricle stroke volume and ejection fraction were reduced in both DOX-treated groups at week six. At the same time as CINE MRI, rats received injections of hyperpolarized pyruvate, and impaired cardiac mitochondrial carbohydrate metabolism was found to assess modifications in real-time in vivo metabolic fluxes. These changes anticipate clinically relevant heart failure (HF) in high-dose treated rats. In addition to the plasma lactate, non-esterified fatty acid (NEFA) and beta-hydroxybutyrate were increased in the high DOX-dose treated at week six. At the end of the last MRI acquisition, the rats’ hearts were excised, and metabolites were extracted for analysis. They found a significant difference between the control group and the treated group: a decrease of the tricarboxylic acid (TCA) TCA cycle-related glutamate, cycle intermediate malate, total carnitine, acetylcarnitine, AMP, ADP, ATP, NAD, phosphocreatine (PCr): ATP ratio in hearts from the control group compared to DOX-treated rats [20].

Finally, at the beginning of this year, Timm’s group published a study based on the metabolic consequences of DOX on rats’ livers, evaluated with metabolomics and hyperpolarized MRI. They looked for liver damage signs or altered liver metabolism. However, they did not find an increase in plasma alanine aminotransferase activity (expression of liver impairment) or changes in liver carbohydrate metabolism in DOX-treated rats, at any time during therapy. Nevertheless, they found an increase in several acyl-carnitine species, as well as rises in citrate, high-energy phosphates, and markers of oxidative stress using metabolomic analysis of liver samples at the end of the therapy. This could be related to premature signs of steatohepatitis, with boosted fatty acids uptake and oxidation, resulting in oxidative stress [21].

Another specific approach to study metabolic alterations was performed by Geng and colleagues, who applied gas chromatography-mass spectrometry (GC-MS) analysis of serum, heart, brain, liver, and kidney to evaluate DOX-toxicity in treated rats. The altered metabolites in the heart were d-lactic acid, 3-methyl-1-pentanol, d-glucose, cholesterol, l-alanine, l-valine, glycerol, glycine, palmitic acid, propanoic acid, phenol and stearic acid. These findings may help to clarify metabolic changes in DOX-treated rats and to understand the mechanisms behind these changes [22]. At the same time, high-resolution nuclear magnetic resonance spectroscopy was used by Gramatyka’s group to identify the early and late signs of ionizing radiation on the murine heart’s metabolism. The hearts of C57Bl/6NCrl female mice were irradiated in vivo with single 0.2 Grey (Gy) or 2 Gy doses; then, tissues were gathered 48 h and 20 weeks after exposure. The most relevant alterations were found 48 h after irradiation, with 2 Gy and comprised high levels of glutamate and pantothenate, as well as reduced concentrations of alanine, malonate, acetylcarnitine, glycine, and adenosine. Twenty weeks after the 2 Gy irradiation, reduced concentrations of acetylcarnitine and glutamine were detected compared to age-matched controls. No statistically significant consequences induced by the 0.2 Gy dose were revealed 20 weeks post-irradiation. The authors also demonstrated that radiation-related effects were not found in the tissue histology, suggesting a higher sensitivity of the metabolomics-based assay in detecting radiation-induced cardiotoxicity [23].

Using the same methodology, Zhou’s group investigated the metabolism of Nintedanib in human and mice liver microsomes to understand the potential toxicity of Nintedanib and to avoid drug-drug interactions when it is co-administrated with other compounds. Several studies have shown that Nintedanib, a TKI used in treating idiopathic pulmonary fibrosis, can cause hepatotoxicity and myocardial damage. Nineteen metabolites were detected in vivo and in vitro metabolism of Nintedanib, and the metabolic pathways primarily included were hydroxylation, demethylation, glucuronidation, and acetylation reactions [24].

Based on the knowledge that dietary polyphenols help to prevent cardiovascular diseases, Lin’s group hypothesized that gut microbiota metabolite regulation might provide cardioprotection, together with the yellow wine polyphenolic compound (YWPC), in DOX-treated rats. The strategy used to modify the microbiota was antibiotics administration in the YWPC group. In 2021, they found that YWPC influences the levels of metabolites altered by DOX (decreased levels of linoleic and arachidonic acid, increased levels of tryptophan) and therefore has a central role in protecting DOX-treated rats against CTX [25].

The latter two animal studies reviewed in this paper focused on the role of the angiotensin receptor blocker CTX prevention. The former recently demonstrated the potential role of Losartan against Sorafenib-induced CTX using a rat model. Sorafenib is an inhibitor of tyrosine kinase (TKI) used in hepatocellular carcinoma and kidney cancer, which may cause CTX. This study demonstrated how Losartan could influence metabolic profile. Some metabolites were associated with CTX, particularly glycin and lactic acid; furthermore, the coadministration of Losartan reverted these changes. In addition, based on histological results, Losartan was able to reverse the cardiac alterations in the cardiac tissue observed in the sorafenib-only treated group [26]. One year later, Alhazzani’s group analyzed the cardioprotective role of Valsartan against DOX-induced CTX using metabolomic and histopathology studies. Sprague-Dawley rats were separated into four groups (control, Valsartan 30 mg/kg, DOX 15 mg/kg and Valsartan in combination with DOX 30 + 15 mg/kg). The combination of Valsartan and DOX resulted in a marked reduction in cardiac biomarker enzymes (creatine phosphokinase and aminotransferase) in comparison with DOX monotherapy; moreover, the histopathological evaluation of the group treated with both Valsartan and DOX revealed less fibrosis and inflammation. The metabolomic analysis of serum metabolites revealed that DOX monotherapy decreased the concentrations of several amino acids; in contrast, combination therapy reversed these metabolic pathways. That is why they support the cardioprotective role of Valsartan [27].

## 4. Step Ahead: The Human Model

### 4.1. In Vitro

It is well known that DOX exhibits cardiotoxicity at cumulative doses, causing increases in intracellular reactive oxygen species in the heart. Spinochrome D (SpD) is a structural analogue of sea urchin pigment echinochrome A. Given that echinochrome A is cardioprotective against DOX-toxicity, at the end of 2018, Chang’s group published a study in which the cardioprotective effects of SpD were tested. They used H-NMR-based metabolomics and mass spectrometry-based proteomics to characterize the metabolites and proteins induced by SpD in a human cardiomyocyte cell line and a human breast cancer cell line. Glutathione metabolism was significantly influenced by SpD treatment in human cardiomyocyte cells, showing that SpD can protect human cardiomyocyte cells from DOX toxicity without affecting the anticancer properties [28].

Subsequently, Palmer’s group developed a metabolic biomarker-based assay to predict the cardiotoxicity potential in human-induced pluripotent stem cell-derived cardiomyocytes (hiPSC-CM). This study was completed in two phases: biomarker identification and targeted assay development. In the first step of the study, four metabolites, related to different metabolic pathways (lactic acid, arachidonic acid, thymidine and 2 ′-deoxycytidine), had essential roles in regulating mitochondrial function, oxidative stress and replication, resulting in their association with CTX [29]. Then, researchers developed a targeted, exposure-based biomarker assay that quantified these metabolites and hiPSC-CM viability across an eight-point concentration curve. Metabolic impairment might help to predict a drug’s potential to cause both structural and functional CTX. Arachidonic acid concentrations were both decreased and increased after cardiotoxic treatment, proving that its metabolism could be affected through multiple pathways. The increase of lactic acid highlights the cell’s decreased capacity to produce ATP via oxidative phosphorylation. Thymidine and 2′-deoxycytidine were both decreased and increased by cardio toxic drugs; the most common response observed was an increase in thymidine levels and a decrease in 2′-deoxycytidine concentrations [29]. This study provides a model that is able to recognize structural and functional cardiotoxic drugs, which could be used to evaluate a drug’s CTX potential comprehensively.

In 2021, Draguet’s group published an in vitro study of metabolic inhibitors through a metabolomic analysis to improve breast cancer treatment. Breast cancer, among others, showed significant fluctuations in choline and glutamine metabolisms. DOX, as stated above, is widely used to treat breast cancer but could be associated with severe cardiac damage. Therefore, they used selected metabolic inhibitors (administered alone or in combination) as potential treatments against breast cancer in order to boost DOX effects. The combination of CB-839 (glutaminase inhibitor) and Oxamate (lactate dehydrogenase inhibitor) and the association of CB-839/Oxamate/D609 (a phosphatidylcholine-specific phospholipase C inhibitor) produced significant cell damage in two breast cancer cell lines (MDA-MB-231 and MCF-7). They were able to increase DOX efficacy in the same cell lines. Those findings reduced patients’ exposure to DOX and avoided severe CTX, while maintaining the same treatment efficacy [30].

Cyclophosphamide, as well as anthracyclines, is an anticancer prodrug that can cause CTX and other severe adverse effects. Recently, Dionisio et al. focused their attention on cyclophosphamide’s CTX using an in vitro metabolomic approach. They suggested a connection between cyclophosphamide’s active metabolite, called 4-hydroxycyclophosphamide, and acrolein, with the toxicity detected after the administration of high doses of cyclophosphamide. Human cardiac proliferative and differentiated AC16 cells were exposed to different concentrations of the three compounds, defining their cytotoxic profile using subtoxic and toxic concentrations for biochemical and morphological studies. Metabolomics was applied to cardiac cells exposed to subtoxic concentrations to identify early damage markers. The results showed that acrolein and 4-hydroxycyclophosphamide prompted mitochondrial and lysosomal impairment with consequent severe cardiotoxicity at significantly lower concentrations than those shown for cyclophosphamide (lower than five µM versus higher than 2500 μM). Acrolein produced higher levels of ATP and total glutathione in proliferative cells at 25 µM. In addition, 4-hydroxycyclophosphamide and acrolein increased sugar levels within the cells, affected some metabolites of the Krebs cycle, and altered amino acid levels. These results support the idea of a dysregulation of catabolism/anabolism equilibrium in proliferative AC16 cells [31].

### 4.2. In Vivo

Different groups have focused their research on the “in vivo” model over the last few years.

Unger’s group proposed, for the first time, a metabolomics-based biomarker panel together with radiation-induced heart damage, using samples from both rat models exposed to radiation and patients receiving radiation therapy for esophageal cancer (Table 2). This study showed metabolic alterations that involved steroid hormone biosynthesis and vitamin E metabolism correlated with cardiac injury. The authors compared plasma profiles of patients who showed CTX after radiation therapy with those who did not, and the results showed lipid dysregulation as a major determinant of cardiac toxicity. They developed a six-metabolite panel which involved SM(d18:1/16:0), SM (d18:1/18:0), PC (16:0/14:0), PE (16:0/20:4), 1-(1,2-Dihexanoylphosphatidyl) inositol-4,5-bisphosphate and Gly-Arg-Gly-Asp-Asn-Pro, all of which were upregulated in plasma of patients who later showed CTX. This study underlines the importance of biomarkers that predict radiation injury in susceptible individuals who may benefit from more robust clinical surveillance [32].

Women affected by breast cancer have been the population most studied. Asnani’s group has recently evaluated the importance of intermediary metabolism in women with breast cancer after anthracyclines and trastuzumab. Analyzing 71 plasma metabolites, the authors recognized variations in aconitic and citric acid that discriminated patients who developed CTX from those who did not. The levels of citric acid at three months were linked with the modification in left ventricular ejection fraction (LVEF) and absolute LVEF at nine months. This study also suggested a perturbation in purine and pyrimidine metabolism in the CTX group. These patients have an increase in the purine metabolites inosine, hypoxanthine, and uric acid, while pyrimidine metabolites pseudouridine and orotic acid matched to those patients who did not show CTX [33].

Once again, studying patients affected by breast cancer, Cocco’s group demonstrated, for the first time, that early anthracycline-induced CTX (detected by asymptomatic Global Longitudinal Strain reduction) is related with a characteristic metabolic profile: in patients with CTX, the authors identified a higher concentration of Krebs cycle intermediates (fumarate and succinate) and fatty acid (such as linoleic acid). On the other hand, patients without CTX instead had increased levels of cardioprotective metabolites, such as tryptophan. Furthermore, cardiotoxicity damage upregulates similar metabolites to those renowned in clinical heart failure and in mice CTX models. These results showed that metabolomics could help to identify patients at high risk of anthracycline-induced CTX, which might benefit a more personalized cardioprotective treatment [34] and other promising strategies [35].

## 5. Conclusions

The discussed studies underline how metabolomics assume greater importance and relevance in cardio-oncology research. There is a growing interest in the research for methods that, associated with clinical and echocardiographic parameters, can allow early identification of patients at higher risk of developing CTX. This engagement would lead clinical practice to a more tailored cardiovascular therapy and a closer follow-up in all CTX-prone patients.

## Figures and Tables

**Table 1:** Summary of techniques and results for early detection of drug-induced cardiotoxicity in animal studies.

Reference	Species	Biofluid/Tissue	Metabolites/Metabolism Discrimination
Andreadu et al., 2009 [4]	Wistar rats	Aqueous myocardial extracts	Increased levels of acetate and succinate, decreased levels of branched-chain amino acids
Andreadu et al., 2014 [5]	Wistar rats	Aqueous myocardial extracts	Perturbations of energy metabolism
Tan et al., 2011 [6]	ICR mice	Myocardial tissue	Increased levels of L-alanine, phosphate, glycine, succinate, malate, proline, threonic acid, glutamine, phenylalanine, dihydroxyacetonephosphate (DHAP), glycerol-3-phosphate (G-3-P), fructose, glucose, stearic acid, myo-inositol and cholesterol; decreased levels of lactate, β-hydroxybutyric acid, l-valine, isoleucine, threonine, citrate, linoleic acid, arachidonic acid
Cong et al., 2012 [7]	Sprague-Dawley rats	Urine	Metabolites involved in metabolic process related to myocardial energy metabolism: tricarboxylic acid cycle (citrate), glycolysis (lactate), pentose phosphate pathway (d-gluconate-1-phosphate) and amino acid metabolism (*N*-acetylglutamine and *N*-acetyl-dl-tryptophan)
Li et al., 2015 [8]	Wistar rats	Plasma	l-carnitine, 19-hydroxydioxycortic acid, LPC (14:0) and LPC (20:2)
Schnackenberg et al., 2016 [9]	B6C3F1 mice	Heart tissue, Plasma	Myocardial specimens: altered levels of 18 amino acids and acetylornithine, kynurenine, putrescine and serotonin, decreased levels of 5 acylcarnitines. Plasma samples: altered levels of 16 amino acids and acetylornithine and hydroxyproline, increased levels of 16 acylcarnitines
Yin et al., 2016 [10]	Wistar rats	Plasma	l-carnitine, proline, 19-hydroxydeoxycorticosterone, phuyoshingosine, cholic acid, LPC (14:0), LPC (18:3), LPC (16:1), LPE (18:2), LPC (22:5), LPC (22:6), linoleic acid, LPC (22:4), LPC (20:2), LPE (18:0), LPC (20:3)
Chaudhari et al., 2017 [11]	Human-induced pluripotent stem cell-derived cardiomyocytes	Culture medium	Reduction in the utilisation of pyruvate and acetate, and accumulation of formate
QuanJun et al., 2017 [12]	BALB/c mice	Serum	DOX administration: increase in 5-hydroxylisine, 2-hydroxybutyrate, 2-oxoglutarate, 3-hydroxybutyrate decrease in glucose, glutamate, cysteine, acetone, methionine, asparate, isoleucine and glycylproline. DZR treatment: increase in lactate, 3-hydroxybutyrate, glutamate, alanine; decrease in glucose, trimethylamine *N*-oxide and carnosine levels
Yun et al., 2021 [13]	C57BL/6 mice	Myocardial tissue	Periplocymarin reduced cardiomyocyte apoptosis protecting myocytes from DOX-induced CTX
Timm et al., 2020 [14]	Wistar rats	Myocardial tissue, Plasma	DOX administration: decrease of the tricarboxylic acid (TCA) cycle intermediate malate, TCA cycle-related glutamate, total carnitine, acetyl carnitine, NAD, AMP, ADP, ATP
Timm et al., 2022 [15]	Wistar rats	Liver tissue	DOX administration: increase in several acyl-carnitine species as well as increases in high energy phosphates, citrate and markers of oxidative stress
Geng et al., 2020 [16]	Sprague-Dawley rats	Serum, heart, liver, kidney, and brain tissue	DOX administration: the altered metabolites in the heart were 3-methyl-1-pentanol, cholesterol, d-glucose, d-lactic acid, glycerol, glycine, l-alanine, l-valine, palmitic acid, phenol, propanoic acid, and stearic acid
Gramatyka et al., 2020 [17]	C57Bl/6NCrl mice	Heart tissue	Ionizing radiation with 2 Gy: high levels of pantothenate and glutamate and decreased levels of alanine, malonate, acetylcarnitine, glycine and adenosine
Zhou et al., 2020 [18]	C57BL/6 J mice	Feces, urine, plasma	Nintedanib metabolic pathways majorly included were hydroxylation, demethylation, glucuronidation, and acetylation reactions
Lin et al., 2021 [19]	Sprague-Dawley rats	Serum	YWPC influences the levels of metabolites altered by DOX (decreased levels of arachidonic and linoleic acid, increased levels of tryptophan)
Abdelgail et al., 2020 [20]	Wistar rats	Serum, heart tissue	Some metabolites were associated with sorafenib-induced CTX, particularly glycin and lattic acid; the coadministration of Losartan reverted these changes
Alhazzani et al.,2021 [21]	Sprague-Dawley rats	Serum	DOX monotherapy reduced concentrations of several amino acids, in contrast the combination therapy reverses these metabolic pathways

**Table 2:** Summary of techniques and results for early detection of drug-induced cardiotoxicity in human studies.

Reference	Species	Biofluid/Tissue	Metabolites/Metabolism Discrimination
Chin Yoon et al., [22]	Human cardiomyocyte cell line (AC 16) and human breast cancer cell line (MCF-7)	Culture medium	Spinochrome D (SpD) influenced glutathione metabolism in AC16 cells and and increased ATP production and the oxygen consumption rate in D-galactose-treated AC16 cells. SpD protected these cells from DOX-induced CTX, reducing the mitochondrial damage of DOX
Palmer et al., 2020 [23]	Human induced pluripotent stem cell-derived cardiomyocytes (hiPSC-CM)	Culture medium	Arachidonic acid, lactic acid, 2′-deoxycytidine and thymidine have important roles in modulating oxidative stress, mitochondrial function and replication resulted associated with CTX
Draguet et al., 2021 [24]	MDA-MB-231 (ATCC^®^ HTB-26TM) and MCF-7 (ATCC^®^ HTB-22TM) cell lines	Culture medium	The combination of CB-839 (glutaminase inhibitor) and Oxamate (lactate dehydrogenase inhibitor) and the combination of CB-839/Oxamate/D609 (a phosphatidylcholine-specific phospholipase C inhibitor) caused significant cell mortality in two breast cancer cell lines (MDA-MB-231 and MCF-7) and were able to improve DOX-efficacy on the same cell lines
Dionisio et al., 2022 [25]	Human cardiac proliferative and differentiated AC16 cells	Culture medium	4-hydroxycyclophosphamide and acrolein induced mitochondrial and lysosomal dysfunction: increased in sugar levels within the cells and a perturbed levels of some metabolites of the Krebs cycle and altered levels of amino acid
Unger et et al., 2020 [26]	Sprague-Dawley rats; Patients receiving radiation therapy for esophageal cancer	Heart Tissue; Plasma	Radiation therapy CTX: SM(d18:1/16:0), PC(16:0/14:0), SM(d18:1/18:0), PE(16:0/20:4), 1-(1,2-Dihexanoylphosphatidyl) inositol-4,5-bisphosphate and Gly-Arg-Gly-Asp-Asn-Pro were upregulated
Asnani et al., 2020 [27]	Women with breast cancer treated with anthracyclines and trastuzumab	Plasma	Changes in citric acid and aconitic acid that differentiated patients who developed CTX
Cocco et al., 2020 [28]	Human population of breast cancer patient	Plasma	In patients with CTX were identified a higher prevalence of Krebs cycle intermediates (fumarate and succinate) and fatty acid (e.g., linoleic acid).

## Abbreviations

CTX	Cardiotoxicity
ATP	Adenosine TriPhosphate
MRI	Magnetic Resonance Imaging
TCA	Tricarboxylic acid
PCr	Phosphocreatine
YWPC	Yellow Wine Polyphenolic Compound
NEFA	Non Esterified Fatty Acids
HF	Heart Failure
GY	Grey
ADP	Adenosine DiPhosphate
AMP	Adenosine MonoPhosphate
AMPK	Adenosine-MonoPhosphate Kinase
NMR	Nuclear Magnetic Resonance
MS	Mass Spectrometry
GC	Gas Chromatography
LC	Liquid Chromatograph
DOX	Doxorubicine
iNOS	inducible Nitric Oxide Synthases
eNOS	endothelial Nitric Oxide Synthases
THP	pirarubicin
L-THP	Liposome pirarubicin
F-THP	Free pirarubicin
UPLC–Q-TOF-MS	Ultra-Performance Liquid Chromatography–Quadrupole Time-of-Flight MS
H-NMR	Hydrogen NMR
hiPSC-CMs	Human induced Pluripotent Stem Cell-Derived CardioMyocytes
DZR	Dexrazoxane
TKIs	Tyrosine Kinase Inhibitors
